# Surface-Based Ultrasound Scans for the Screening of Prostate Cancer

**DOI:** 10.1109/OJEMB.2024.3503494

**Published:** 2024-11-20

**Authors:** Rory Bennett, Tristan Barrett, Vincent J. Gnanapragasam, Zion Tse

**Affiliations:** School of Engineering and Materials ScienceQueen Mary University of London 4617 E1 4NS London U.K.; Department of Radiology, Addenbrooke's HospitalUniversity of Cambridge School of Clinical Medicine 12204 CB2 0QQ Cambridge U.K.

**Keywords:** Abdominal ultrasound, machine learning, prostate cancer, PSA-density, surface-based ultrasound

## Abstract

Surface-based ultrasound (SUS) systems have undergone substantial improvement over the years in image quality, ease-of-use, and reduction in size. Their ability to image organs non-invasively makes them a prime technology for the diagnosis and monitoring of various diseases and conditions. An example is the screening/risk- stratification of prostate cancer (PCa) using prostate-specific antigen density (PSAD). Current literature predominantly focuses on prostate volume (PV) estimation techniques that make use of magnetic resonance imaging (MRI) or transrectal ultrasound (TRUS) imaging, while SUS techniques are largely overlooked. If a reliable SUS PCa screening method can be introduced, patients may be able to forgo unnecessary MRI or TRUS scans. Such a screening procedure could be introduced into standard primary care settings with point-of-care ultrasound systems available at a fraction of the cost of their larger hospital counterparts. This review analyses whether literature suggests it is possible to use SUS-derived PV in the calculation of PSAD.

## I. Introduction

Prostate cancer (PCa) is the second most diagnosed form of cancer in men, with a mortality rate second only to lung cancer [1]. It has a five-year survivability rate that drops from nearly $100\% $ to $32\% $ in those diagnosed with stage 3 and stage 4 cancer, respectively [2]. This dramatic decrease underscores the importance of earlier detection.

A variety of methods are available for detecting PCa, each with their own advantages and disadvantages, from the simple digital rectal exam (DRE), through to automatic segmentation of multi-parametric magnetic resonance images (mpMRI) leveraging deep learning. This review focuses on detecting PCa by combining the result of a prostate-specific antigen (PSA) blood test with prostate volume (PV) measurements, acquired using surface-based ultrasound (SUS), to give PSA-density (PSAD) [3].

The calculation of PSAD is shown in (1), where PSA is in ${ng/mL}$, PV is in $mL$, and the resulting PSAD is in ${ng/m{{L}}^2}$. The benefit of using PSAD over PSA is that it accounts for enlarged prostates naturally increasing PSA levels in the blood, which alone is not necessarily a sign of PCa.

\begin{equation*}
PSAD = \frac{{PSA}}{{PV}} \tag{1}
\end{equation*}

It has been shown that PSAD is more reliable than PSA alone when attempting to detect PCa in patients with a Gleason Score of $7$ or higher [6] and can be used to safely avoid biopsies in patients with negative features on magnetic resonance images (MRI) [7], [8].

**Fig. 1.**
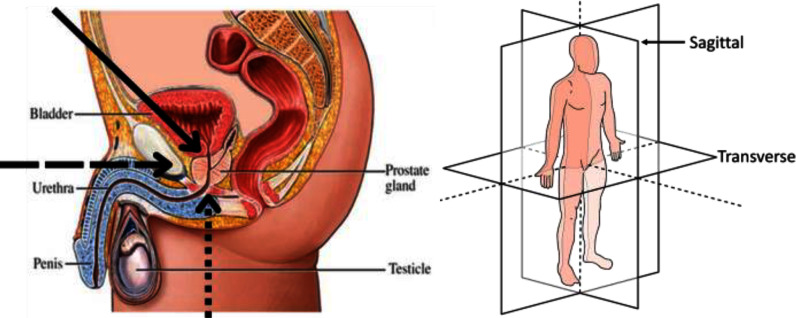
Left – male abdominal diagram showing prostate gland location. Arrows indicate SUS viewing angles (solid and dotted – suitable; dashed – unsuitable) [4]. Right –anatomical planes [5].

Acquiring PV for use in PSAD calculations can be tricky. The difficulty in acquiring the PV is in part due to there being no orthogonal line-of-site from outside the body to the prostate that is not at least partially obstructed by bone (see Fig. 1– Left, dashed and dotted arrows). While not a problem for MRI, it is a problem for SUS imaging.

**Fig. 2.**
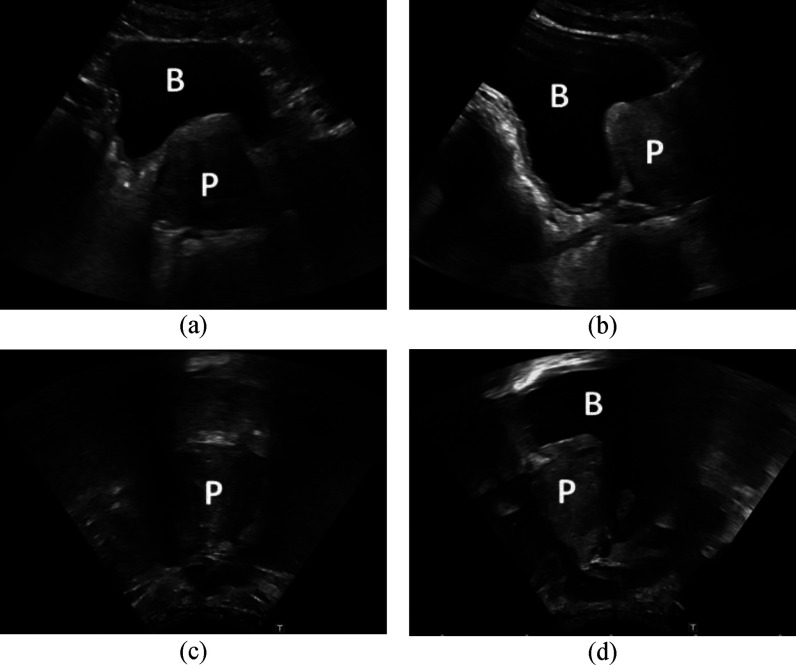
A comparison between the different SUS images of the prostate. (a) – tAUS scan. (b) – sAUS scan. (c) – tTPUS scan. (d) – sTPUS scan. Each image was taken to be at the centre of the prostate. ‘B’ indicates the bladder and ‘P’ the prostate.

SUS scans of the prostate can be split into: Transabdominal ultrasound (AUS) scans and transperineal ultrasound (TPUS) scans. For AUS scans the probe is placed on the abdomen of the patient (see Fig. 1– Left solid arrow) whereas for TPUS scans the probe is placed on the perineum (see Fig. 1– Left dotted arrow). Each of these scanning positions are further subdivided into two orthogonal sets: transverse and sagittal. For each of these scans the ultrasound probe is aligned with the relevant anatomical plane in Fig. 1– Right. This results in four possible scanning planes: transverse-AUS (tAUS), sagittal-AUS (sAUS), transverse-TPUS (tTPUS), and sagittal-TPUS (sTPUS). Fig. 2 highlights the differences between AUS scans (top) and TPUS scans (bottom) of the prostate.

**Fig. 3.**
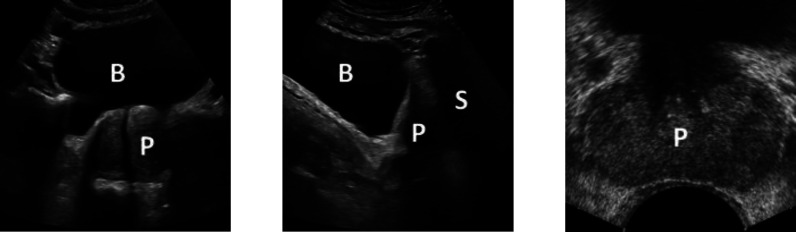
An example of a tAUS scan (Left), sAUS scan (Centre), and TRUS scan [9] (Right) of the prostate. ‘B’ indicates the bladder, ‘P’ the prostate, and ‘S’ shadowing caused by the pubic bone. The tAUS and sAUS images were acquired using a Canon Aplio i700 ultrasound system.

SUS scans are not subject to the patient discomfort of transrectal ultrasound (TRUS) scans or operating constraints of mpMRI scans. However, due to SUS having a lower signal-to-noise ratio compared to that of TRUS, SUS images containing multiple anatomical structures (see Fig. 3 – Left and Centre), and the shadowing caused by the pubic bone (Fig. 1– Left solid arrow and resultant shadowing in Fig. 3 – Centre), there is comparatively little research into the acquisition of PV using SUS images.

The current review covers the use of SUS scans in acquiring PV estimates, considering its suitability for use in PSAD calculations when testing for PCa. A summary of the studies reviewed is presented in Table 1, with some key metrics given in Fig. 5. The methodology followed can be found in the supplementary materials, as can the recognised limitations of this study.

**TABLE 1:** Paper Search Results, With Key SUS Conclusions, Organised by Year Published

Ref.	Focus	Cohort Size	SUS Position	Equation	Reference	Over/Under/Accurate (SUS w.r.t. reference PV)	Correlation (SUS w.r.t. reference)	Stratification	SUS Conclusions
[16]	PV	15	AUS	PE	Specimen	-	-	-	Useful applications where approximate assessments are required.
[17]	PV	8	AUS	Sphere	Specimen	-	•	-	AUS is more accurate than CT and clinical estimates in PV estimation.
[29]	PV	29	AUS	Sphere	Specimen	-	Highly significant correlation. • $r = .95,\ p = .0005.$	Better correlation for larger prostates.	AUS should receive more attention as it is an accurate predictor of PV.
[38]	PV	50	AUS	PE	Specimen	-	Correlation. • $r = .988$	-	AUS is an accurate method for estimation of PV.
[44]	PV	33	AUS	PE	Specimen	-	• Insignificant difference.	-	AUS is reasonably accurate with good correlation to specimen weight.
[45]	PV	85	AUS	-	Specimen	-	-	-	Close correlation between AUS and sum of resected and postoperative PV.
[27]	PV	26	AUS	PE	Specimen	Underestimated.	Pearson's correlation. • $r = .86.$ Spearman's correlation. $ \bullet \ r = .76.$	-	AUS is quite accurate in PV estimation has definite value in the assessment of prostatic size.
[46]	PV	88	AUS	-	TRUS	-	• Correlated well with good agreement.	-	AUS correlated well with TRUS.
[39]	PV	107	AUS	Sphere	Specimen	-	Pearson's correlation. $ \bullet \ r = .956.$	-	AUS was well adapted for screening of prostatic diseases.
[30]	PV	50	TPUS	PE^1^	TRUS	-	Student's two-tailed t-test. • $r = .876.$	-	TPUS measurements are comparable to TRUS in dimensions and volume.
[40]	PV	80	TPUS	PE	Specimen	-	Pearson's correlation to TPUS. • Specimen: $r = .89,\ p < .0001.$ • AUS: $r = .92,\ p < .0001$.	-	TPUS is accurate in evaluating PV.
[41]	PV	44	AUS	PE	TRUS	-	Pearson's correlation. • $r = .82.$	-	No statistically significant difference between AUS and TRUS.
[42]	PV	95	AUS	PE	TRUS	-	Pearson's correlation. • $r = .948,\ p < .001.$	-	AUS could replace TRUS for PV determination.
[23]	PV	196	AUS	PE	TRUS	Overestimated: $45.9\% .$ Underestimated: $26.5\% .$ Accurate:$\ 27.5\% $.	-	-	AUS less accurate than TRUS
[47]	PSAD	420	AUS	PE^5^	Biopsy	-	PSAD ROC curve areas. • TRUS: $.66.$ • AUS:$\ .67.$ Sensitivity and specificity. • TRUS: $.77$ and $.4.$ • AUS: $.75$ and $.49$.	-	AUS derived PSAD is as useful as TRUS derived PSAD.
[31]	PV	22	AUS	PE	TRUS	-	Spearman's correlation^4^. • 100mL: $.77.$ • 200mL: $.65.$ • 300mL: $.69.$ • 400mL: $.58.$ • 500mL: $.46.$	-	AUS PV correlates well with TRUS PV when bladder volume $ < \ 400mL$.
[32]	PV	200	AUS	PE	TRUS	-	Pearson's correlation. $ \bullet \ r = .84,\ p < .001.$	-	No statistical difference between AUS PV and TRUS PV.
[48]	**ML**	**11**	**AUS**	**-**	**-**	**-**	• **-**	**-**	**-**
[22]	PV + PSAD	238	AUS	PE^2^	TRUS	Overestimated: $38.2\% .$ Underestimated: $31.5\% .$ Accurate: $30.3\% $.	Pearson's correlation (PV). • All:$\ r = .799,\ p < .0001.$ • >50mL: $r = .756,\ p < .0001.$ • <50mL:$\ r = .481,\ p < .0001.$	Better correlation for larger prostates.	AUS PSAD is worthwhile as it significantly improves on PSA alone and can reduce unnecessary biopsies.
[43]	PV	287	TPUS	PE	TRUS	-	Interclass correlation (PV). • $.92\ ( {.9 - .94} )$	-	TPUS provides an accurate alternative to TRUS.
[25]	PV	94	AUS	PE	TRUS	Overestimated:$\ 37\% .$ Underestimated $59\% .$ Accurate: $4\% $.	Pearson's correlation. • All:$\ r = .775,\ p < .01.$ • Beginner:$\ r = .408,\ p < .01.$ • Trained: $r = .7,\ p < .05.$ • Expert:$\ r = .967,\ p < .05$.		-
[19]	PV	100	AUS	PE	TRUS	Overestimated by $5.47\% $.	Pearson's correlation. • All:$\ r = .94,\ p < .001.$ • >50mL: $r = .9,\ p < .001.$ • <=50mL:$\ r = .77,\ p < .001.$	Better correlation for larger prostates.	Strong correlation between AUS and TRUS for volume and dimensions. AUS can be an alternative to TRUS.
[20]	PV	71	AUS	PE	Specimen	Overestimated by $55.7\% .$	Pearson's correlation. • $r = .818,\ p < .0005$.	-	AUS shows significant overestimation of specimen weight.
[21]	PV + PSAD	60	AUS	PE	Specimen	Overestimated.	Pearson's correlation. • PV: $P = .03$. • PSAD: $P = .28$.	-	AUS PSAD showed no statistically significant difference to specimen PSAD. AUS overestimation of specimen volume statically significant.
[24]	PV	163	AUS	PE	Specimen	Underestimated by $8.2\% $.	95% confidence interval. • $.838\ ( {.774--.883} )$	Most accurate estimation between 41g-60g..	AUS PV correlated well with specimen.
[33]	PV	40	AUS	PE	TRUS	-	Pearson's correlation. • $r = .99,\ p < .001.$	-	AUS does not agree sufficiently with TRUS according to LOA.
[34]	PV	60	AUS	NR	Specimen	-	Pearson's correlation. • r=.77, p<.001. Coefficient of linear regression. $ \bullet \ .127,\ p = .641.$	-	Significant correlation between AUS and specimen.
[35]	PV	49	AUS	PE	TRUS	-	Spearman's correlation. • $r = .838,\ p < .01.$	-	TRUS and AUS are not different in PV estimation.
[49]	**ML**	**210**	**AUS**	**-**	**-**	**-**	**-**	**-**	**-**
[28]	PV	236	AUS	PE	TRUS	Mix of overestimation, underestimation, and accurate estimation.	Pearson's correlation. • $r = .87.$ Intraclass correlation. • $.93\ ( {.91 - .95} ).$	Better agreement^5^ for smaller prostates.	AUS excellent surrogate for TRUS in most cases, but not for larger prostates.
[18]	PV	170	AUS	PE	Specimen	Overestimated.	Spearman's correlation. • $r = .776,\ p = .0001\ sig\ 2\ tailed\ 0.0.$	-	Positive correlation between AUS and specimen.
[36]	PV	92	AUS	PE	Specimen	-	Pearson's correlation. • $r = .784,\ p < .001.$	-	AUS showed statistically significant difference to specimen.
[26]	PV	98	AUS	PE	Specimen	Overestimated: $10\% $. Underestimated: $66\% $. Accurate: $24\% $	Pearson's correlation. • All: $r = .877,\ p < .001.$ • <30g: $r = .352\ p = .078.$ • 30g-60g: $r = .299,\ p = .033.$ • >60g: $r = .911,\ p < .001$	Better correlation for larger prostates.	AUS comparable, and acceptable alternative, to TRUS.
[50]	**ML**	**305**	**AUS**	**PE**	**-**	**-**	**-**	**-**	**-**
[37]	PV + PSAD	64	AUS / TPUS	PE	mpMRI	-	Interclass correlation (PV). • TAUS to MRI: $.87\ ( {.8--.92} ).$ • TPUS to MRI: .$78\ ( {.65--.86} ).$	-	AUS and TPUS PV has good agreement with MRI PV. AUS and TPUS PSAD has good agreement with MRI PSAD.

Bolded Rows Attempted to Automate All/Part of the PV Estimation Process. PE – Prolate Ellipsoid From (2). PE^1^ – Modification of (2): $( {\pi W \wedge 2\ L} )/6 < 80g$ or $( {\pi W \wedge 3} )/6 > 80g$. PE^2^ – Modification of (2): $( {\pi W \wedge 2\ L} )/6$. PE^3^ – Modification of (2): ${{{\bm{L}}}^2}{\bm{W}}/2$. Spearman's Correlation^4^: With Respect to Bladder Volume. Agreement^5^: As Determined Using Bland Altman Plots and Limits-of-Agreement

## II. Review Search Results

The results of the literature search can be categorised into four groups: How PV is estimated using SUS scans; The accuracy of PV estimates calculated using SUS images; How SUS-derived PSAD values fare in the clinical decision process in comparison to more contemporary methods; Automating the process of PV estimation using SUS images.

### A. SUS-Based PV Estimation

In a clinical setting, there are generally two approaches used to estimate the PV from images: stepwise planimetry and geometric models. To the best of the authors’ knowledge, there are no studies done to date that use SUS images of the prostate for stepwise planimetry, and as such, only the geometric model is considered.

The geometric model assumes that the prostate is shaped like an ellipse. This is not entirely true, and as patients age the prostate can become more irregularly shaped. Under this assumption (2) is used to estimate PV, where $L$, $W$, and $H$ are the length, width, and height of the gland, respectively, and C is a constant. For the sake of this review and the equations presented in Table 1, $L$ is the anterior-posterior dimension, $W$ is the left-right dimension, and $H$ is the superior-inferior dimension. The value of the constant C varies based on the assumed shape of the prostate [10], [11], [12], [13].

\begin{equation*}
PV = L \times W \times H \times C \tag{2}
\end{equation*}

**Fig. 4.**
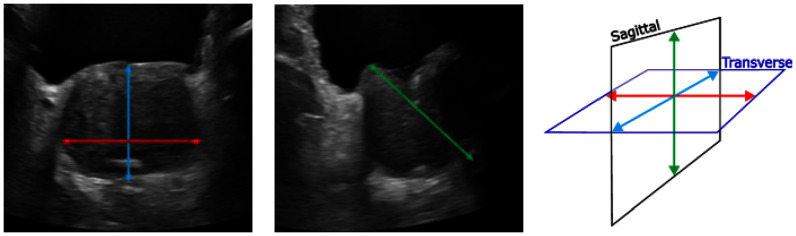
AUS measurement locations used to estimate PV. Left - transverse plane showing axial 1 and axial 2 measurements. Middle - sagittal plane showing cranio-caudal measurement. Right – anatomical scan planes with corresponding dimensions superimposed.

Two orthogonal images of the prostate are acquired, and a clinician measures the three required dimensions. See Fig. 4 showing sample measurements on both a tAUS (Left) and sAUS (Middle) image. Studies tend to conclude that the geometric model is accurate enough when compared to the more accurate stepwise planimetry [14], [15].

### B. SUS-Based PV Accuracy

The use of AUS scans to estimate PV was mentioned as far back as 1973 [16]. It was concluded that the SUS-derived PV estimates were useful in applications that required approximate assessment of the prostate. In 1977, [17] concluded that SUS-derived PVs were more accurate than CT-derived and clinically derived values when compared to specimen weights, and that SUS could be used when estimating PV.

Since then, multiple studies have tested the accuracy of SUS-derived PV estimates in comparison to one or more reference values. Some found that SUS-derived PVs tended to overestimate the reference method [18], [19], [20], [21], [22], [23], others showed a tendency to underestimate [24], [25], [26], [27], while some found a mixture of underestimation, overestimation, and accurate estimation [22], [23], [25], [26], [28]. In attempts to determine if measurements of smaller or larger prostates using SUS scans correlated more with the reference method some studies stratified their results based on volume estimates. References [19], [22], [26], [29] showed better correlation with larger prostates, [28] showed more agreement for smaller prostates, and [24] showed the best correlation for medium sized prostates. Of the 31 studies that compared SUS-derived PV with a reference value, quantitatively 24 of them found that SUS-derived PV estimates correlated well with the chosen reference method [18], [19], [20], [22], [24], [25], [26], [27], [28], [29], [30], [31], [32], [33], [34], [35], [36], [37], [38], [39], [40], [41], [42], [43], with six studies not reporting a quantitative correlation value [16], [17], [23], [44], [45], [46]. One study did not report a correlation coefficient, only that the SUS-derived PV significantly overestimated the reference PV [21].

Of these 24 studies, 5 concluded that even with the reported correlation values SUS-derived PV is not suitable when compared to the chosen reference method [20], [21], [23], [33], [36]: Three studies concluded that SUS-derived PVs overestimated specimen weight [20], [21], [23]; One study explicitly claimed that SUS could not be used in place of TRUS, stating that SUS-derived PV estimates did not agree with TRUS-derived PV estimates using Bland Altman plots and limits-of-agreement [33]; One study concluded that there was a statistically significant difference between SUS-derived PV and specimen weight [36].

### C. Clinical Decisions

A 2002 study compared SUS-derived and TRUS-derived PSAD values with the results of a biopsy [47], with the conclusion being that SUS-derived PSAD is as useful as TRUS-derived PSAD.

In a 2005 study PSAD values calculated using SUS-derived and TRUS-derived PV estimates were compared [22]. Although it was found that SUS-derived and TRUS-derived PVs correlated well, the SUS-derived PSAD values did not perform as well as the TRUS-derived values in detecting PCa. However, it was noted that SUS-derived PSAD values significantly outperformed PSA alone.

A 2013 study showed that due to SUS-derived PV estimates tending towards overestimation of the specimen volume, $41.7\% $ of cancers would have been missed, in their cohort, if SUS-derived PSAD values were used for PCa prediction [21]. This was in comparison to the $30\% $ that would have been missed if specimen volumes were used in the calculations of PSAD values. The study concluded that SUS-derived PSAD values performed similarly to TRUS-derived values for active surveillance.

In a more recent study (2022) PSAD values derived from SUS scans were compared with MRI-derived PSAD values [37]. The aim of the study was to ascertain whether SUS-derived PSAD values could be used in triage as a risk-stratification tool. A more conservative PSAD threshold was used with sensitivities and specificities of up to $100\% $ achieved. It was concluded that SUS-derived PSAD has a good agreement with MRI-derived values. They also suggested that unnecessary MRIs can be avoided by using the PSAD values obtained from SUS scans.

### D. Automating SUS-Based PV Estimation

A 2004/5 study attempted to automatically segment AUS images of the prostate using a combination of a contour enhancing filter and a heuristic optimisation algorithm [48]. They found that their system produced contours that were very similar to those created through manual segmentation.

In 2017 [49] and 2022 [50] a multi-task quadruplet deep convolutional neural network (QDCNN) was developed to infer four points on tAUS images of the prostate, and two points on sAUS images. These six points were then used to estimate the PV using (2) (see Fig. 2 of the Supplementary Material for sample results). It was found that the QDCNN system's estimated PVs fell within experts’ estimations, and that the system could be used by experts as an aid to increase the reliability of their own PV estimates.

## III. Discussion

While SUS PV estimation is not a new idea its limitations have resulted in the technique largely being ignored in favour of other imaging modalities, which are considered superior when imaging the prostate. However, they are subject to higher operating costs, increased time requirements, and patient discomfort. SUS scans are not subject to any of these limitations, with point-of-care ultrasound (POCUS) devices allowing for routine scanning in a primary care setting. SUS scans exhibit almost no patient discomfort and are non-invasive, however, the images are affected by shadowing from the pubic bone. This shadowing can lead to further inaccuracies in the PV estimates as the prolate ellipsoid dimensions can be difficult to estimate. To minimise the effects of this pubic bone shadowing, patients are asked to present with a full bladder (see Fig. 3 of the Supplementary Material). If SUS systems can be shown to be good enough for PCa detection purposes, they could be used as a pre-MRI/TRUS scan, or for use during active surveillance, and potentially for the screening of PCa. For this to be realised, SUS-derived PV estimates need to be shown to correlate well with either mpMRI, TRUS, or specimen volumes, but more importantly the resulting PSAD values need to be shown as effective in detecting PCa.

When manually estimating the PV simpler methods/ techniques are favoured by clinicians due to time constraints and ease of use. Generally, the higher the required accuracy the more complicated/time-consuming the estimation method will be (manual stepwise planimetry versus geometric models). Manual stepwise planimetry of mpMRI has been suggested by some to be used as the gold-standard, whereas prolate ellipsoid PV estimates are just considered good enough or reasonably accurate [14], [51]. The drop in accuracy between manual planimetry and the prolate ellipsoid formula can be attributed to the limitations associated with using a simple geometric model to represent the prostate, which can have a variety of shapes that tend to change as the patient ages [12], [13]. This variability in prostate shape suggests that the constant value of the geometric model may not be the most accurate formulation, and that instead a variable constant (possibly a function of the prostate size) should be used. Such a formula has been reported previously, where the “adjusted PV” is a function of patient age and the calculated prolate ellipsoid volume of the prostate [36].

**Fig. 5.**
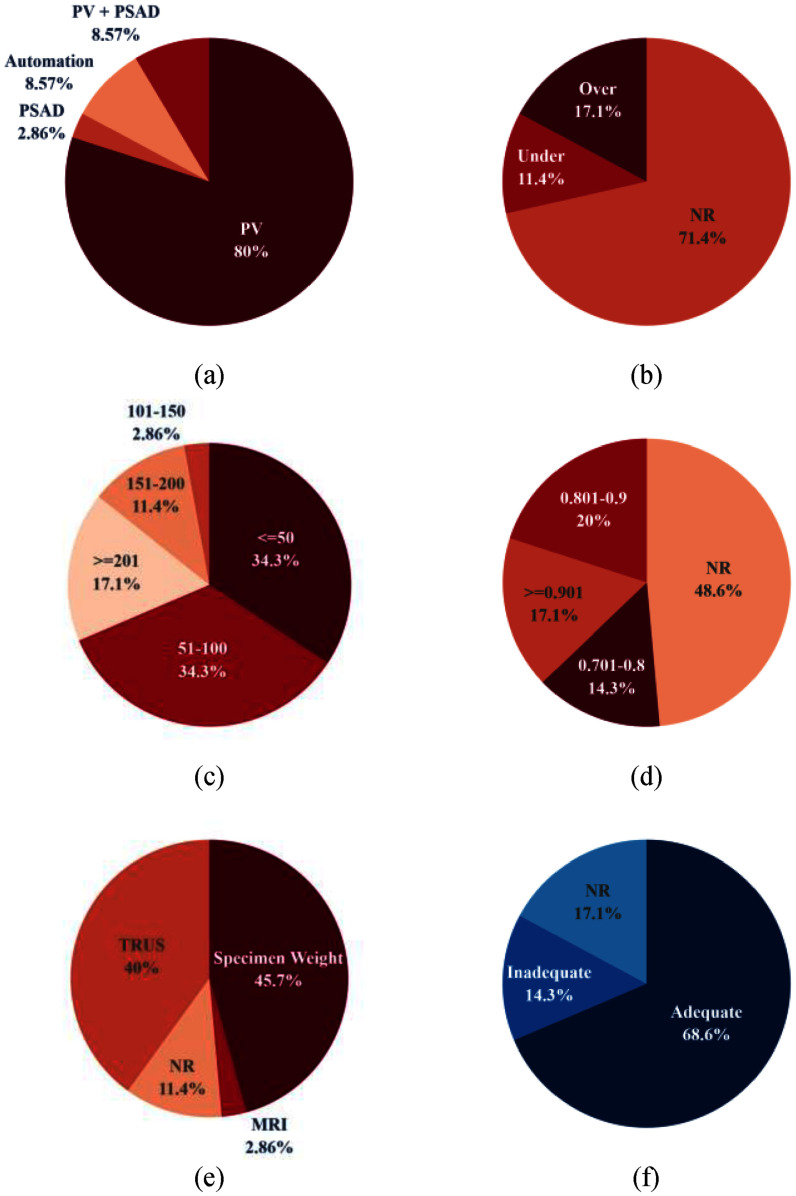
Graphical summary of key metrics from Table 1. NR – Not report or not applicable. (a) Focus of paper. (b) Overestimation or underestimation of SUS-derived PV w.r.t. reference method. (c) Study cohort size, grouped by 50. (d) Reported PCC. (e) PV estimation reference method. (f) Qualitative conclusion regarding suitability of AUS in PV estimation.

While the absolute accuracy of SUS-derived PV estimates may vary between studies the general consensus is that they are fairly accurate when compared to either mpMRI, TRUS, or specimen weight (Fig. 5(f)). Of the five studies that found AUS-derived PV estimates were significantly different from their chosen reference method, only one explicitly claimed that AUS-derived PV estimates could not be used in place of TRUS-derived estimates [33]. However, in a later study, it was noted that the cohort of the 2015 study was relatively small and homogeneous [28]. Fig. 5(d) highlights the strong correlation between SUS-derived PV values and the chosen reference method for the papers included in this review. When reported whether SUS-derived PV was overestimated, underestimated, or accurately estimated, most studies found overestimation to be more common (Fig. 5(b)).

Studies that have used SUS-derived PSAD values to detect PCa show that even though the absolute accuracy of geometric SUS-derived PV estimates may not always be very reliable, the resulting PSAD values are accurate enough to be used in the process of detecting, and possibly screening for, PCa [21], [22], [37], [47]. The results of [37] are particularly promising for SUS PCa screening. By simply lowering the threshold PSAD value they were able to reach a sensitivity of $100\% $ for SUS-derived PSAD values. Although PVs estimated from SUS scans have been shown to not be entirely accurate, they are consistent enough that merely changing other parameters (not related to the acquisition of the PV) to accommodate this lack of accuracy is sufficient.

In comparison to MRI- and TRUS-based automated PV estimation studies, there are not many that use SUS scans. The three studies presented are the only studies that work towards automatically estimating PV from AUS-based scans, with [50] a continuation of [49]. The first of these two studies did not attempt to estimate PV as only the first two dimensions of (2) were inferred from tAUS images. The second study improved on this limitation by incorporating inference of the third dimension from sAUS images. [48] only attempted to segment one image of the prostate, and no volume calculations were attempted. The presented results of [50] are encouraging as the level of accuracy their system was capable of fell within those of expert values. They also made their dataset available for public use, which helps address a major bottleneck researchers face when creating new machine learning models: data availability. When attempting to create systems that can automate the task of estimating PV from SUS scans acquiring datasets for a single study can be an expensive and protracted process. Public datasets alleviate this and can grant researchers access to a more diverse pool than when using their own datasets. Fig. 5(c) shows the limited cohort sizes in the studies presented. Only six studies had more than $200$ patients participate, with most studies making use of data from less than $100$ patients.

## IV. Conclusion

When it comes to testing for PCa using PV and PSAD the most trusted imaging modality is mpMRI. This is followed by TRUS which is considered invasive. SUS comes in last, due to its lower signal-to-noise ratio, and the presence of multiple confounding anatomical structures in its images. However, the results of this review show that even though SUS-based PV estimates are not the most accurate, they are accurate and consistent enough that they can be used for the calculation of PSAD in a clinically appropriate setting.

Due to the known limitations of SUS scans of the prostate there is comparatively little research into using machine learning to either aid in, or fully-automate, the process of calculating the PV. However, the few studies that have attempted to automate the process of calculating PSAD (by first automating the process of estimating PV) using machine learning have shown very promising results.

Therefore, given that SUS scans of the prostate return “good enough” PV estimates with significant correlation to more accurate methods, and machine learning has been shown to be capable of returning results within the range of experts, further investigation into the use of SUS scans of the prostate for the screening of PCa is definitely warranted.

## Supplementary Materials

Supplementary materials have been supplied as a separate file. The methodology followed during the literature search, some sample images, and a limitations section can be found there.
